# Supplementary material for: Subgrapher: visual fingerprinting of chemical structures
Source: J Cheminform. 2025 Sep 29;17:149. doi: 10.1186/s13321-025-01091-4 (PMC12482248; doi:10.1186/s13321-025-01091-4)
Supplement: Supplementary file 1 — (pdf 3861 KB) [file 13321_2025_1091_MOESM1_ESM.pdf]

# Supplementary Information: SubGrapher: Visual Fingerprinting of Chemical Structures

Lucas Morin[1, 2] Gerhard Ingmar Meijer[1] Valéry Weber[1]
Luc Van Gool[3, 2] Peter W. J. Staar[1]

[1] IBM Research, Säumerstrasse 4, 8803 Rüschlikon, Switzerland
[2] Department of Information Technology and Electrical Engineering, ETH Zurich, Sternwartstrasse 7, 8092 Zürich, Switzerland
[3] INSAIT, Sofia University St. Kliment Ohridski, Tsarigradsko shose 111R, 1784 Sofia, Bulgaria

# Supplementary Note 1

To assess the coverage of our substructures, we analyzed their presence in 122M molecules from PubChem. First, we evaluated how well our functional groups cover the heteroatoms in molecules that contain at least one heteroatom. We found that 97% of such molecules include at least one of our functional groups. On average, 77% of their heteroatoms are covered by at least one detected functional group. Second, we performed the same analysis for carbon backbones in molecules composed solely of carbon atoms. We found that 95% of these carbon-only molecules include at least one of our carbon patterns. On average, 71% of their atoms are captured by at least one carbon backbone from our list.

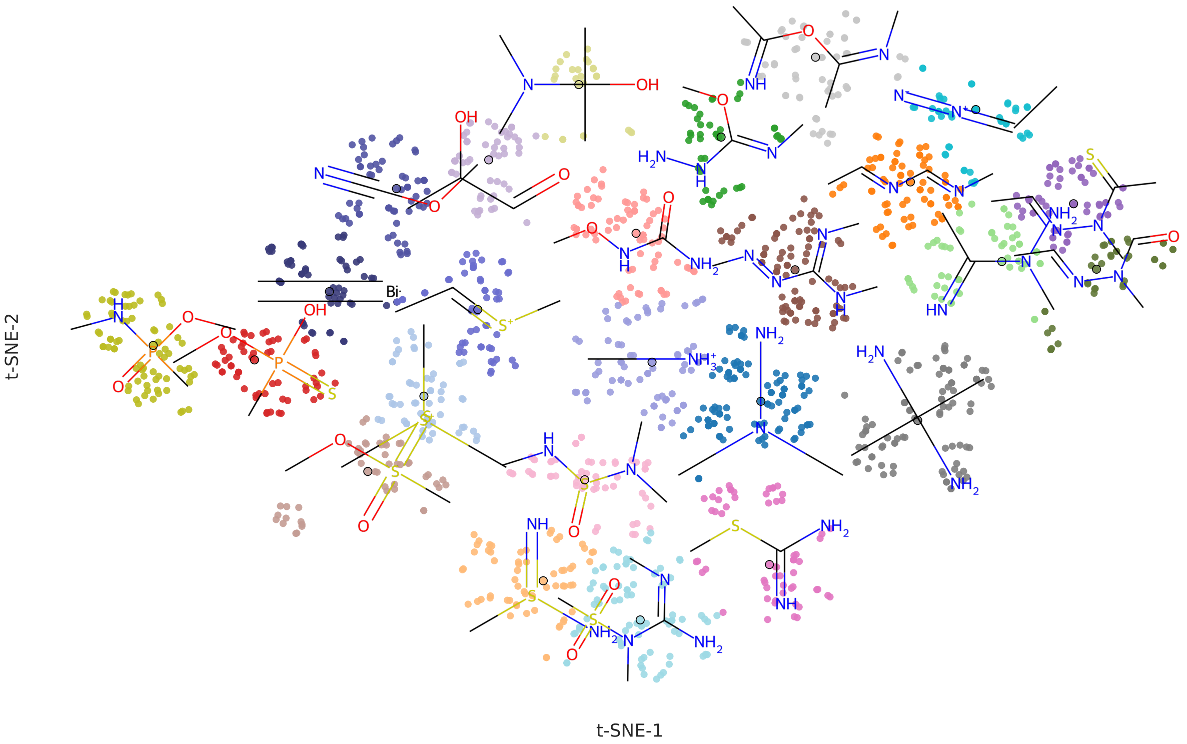


**Supplementary Figure 1**: **Functional groups diversity.** t-SNE visualization of 25 functional group clusters. Each cluster is represented by colored points and overlaid with the centroid molecule.

| Expansion value | Adenosine | Camphor | Cholesterol | Limonene | Pyridine |
|---|---|---|---|---|---|
| $-1 * d$ | 125 | 80 | 126 | 89 | 112 |
| $0 * d$ | 111 | 74 | 106 | 82 | 105 |
| $0.05 * d$ | 111 | 74 | 106 | 81 | 103 |
| $0.1 * d$ | 110 | 73 | 106 | 81 | 103 |
| $0.25 * d$ | 110 | 73 | 106 | 82 | 104 |

**Supplementary Table 1**: **Expansion value sensitivity analysis.** We compare the retrieval performance of SubGrapher for various expansion values of the detected bounding boxes on image datasets generated from adenosine, camphor, cholesterol, limonene, and pyridine. We report the average rank at which a query molecule is retrieved across 50 queries. $d$ denotes the diagonal length of the smallest detected box in the image.

| Fingerprint | Type | Dimension | Adenosine | Camphor | Cholesterol | Limonene | Pyridine |
|---|---|---|---|---|---|---|---|
| RDKit [1] | Binary | $1 \times 4096$ | 1465 | 392 | 696 | 230 | 249 |
| HMFP [2] | Integer | $1 \times 2048$ | 2048 | 2048 | 2048 | 2048 | 2048 |
| **SVFP (Ours)** | Float | $1561 \times 1561 =$ $1 \times 2436721$ | 43 | 20 | 35 | 15 | 7 |

**Supplementary Table 2**: **Fingerprints characteristics comparison.** Comparison of fingerprints types, dimensions, and average number of non-zero coefficients computed on image retrieval benchmarks generated from adenosine, camphor, cholesterol, limonene, and pyridine. For RDKit and HMFP, the fingerprints are obtained using OSRA predictions.

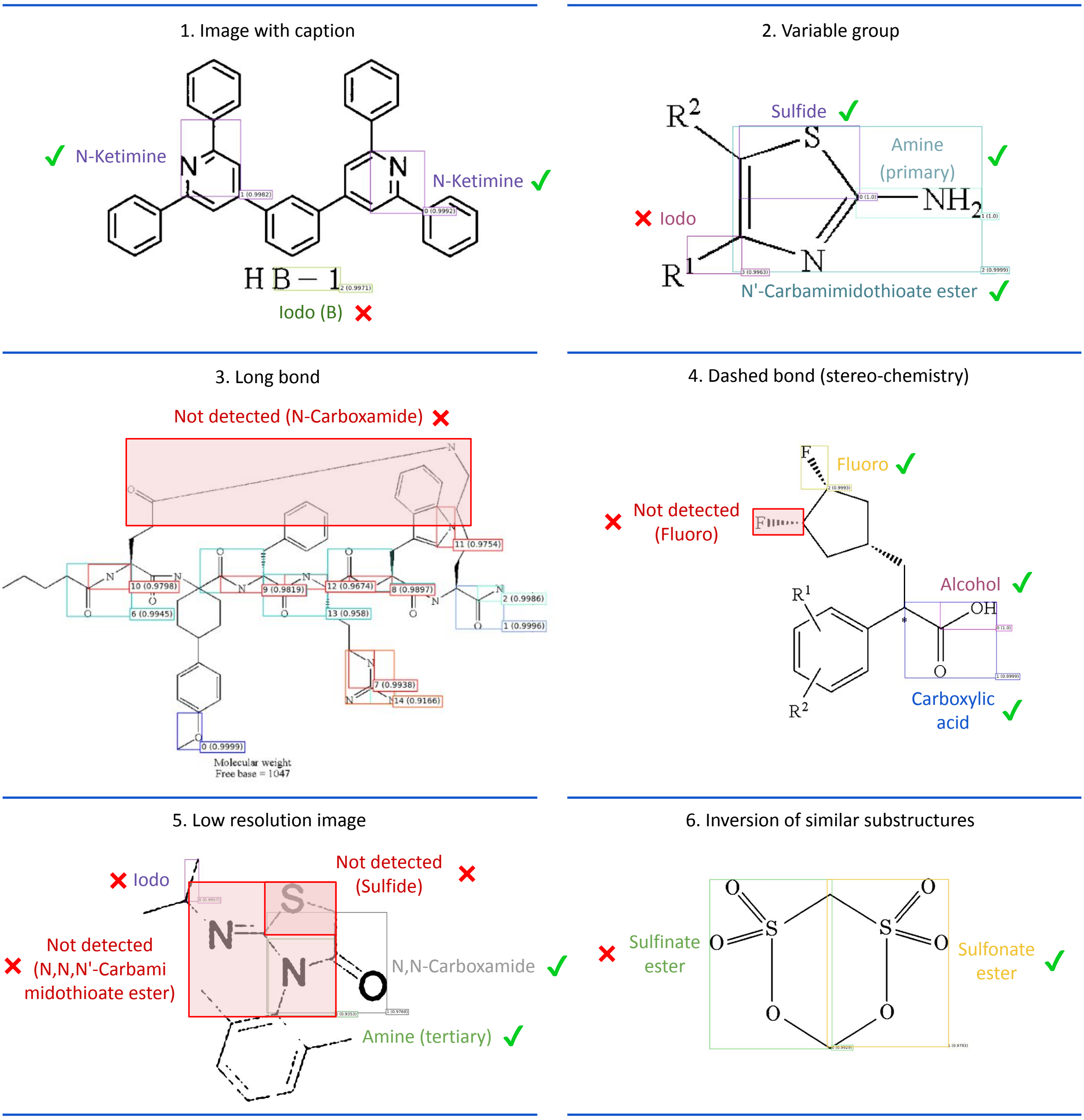


**Supplementary Figure 2**: **Failure cases.** Example of failure cases of SubGrapher on real-world data from JPO, USPTO-Markush and USPTO-10K-L. Typical failure cases include images containing captions (input 1), variable groups (input 2), long bonds (input 3), dashed bonds indicating stereo information (input 4), low resolution images (input 5) and inversions of similar substructures (input 6).

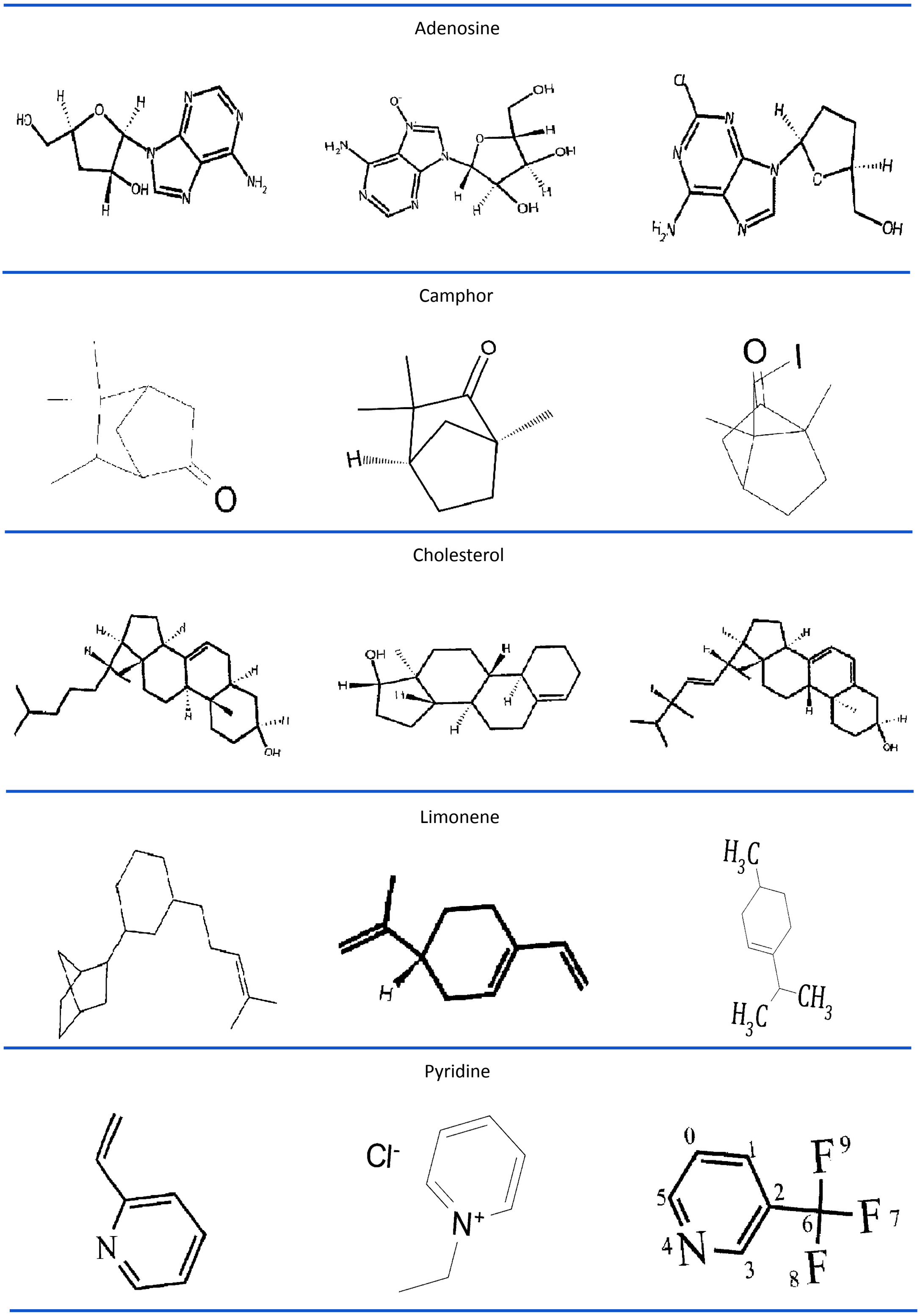


**Supplementary Figure 3**: **Benchmarks example images.** Example images randomly selected from the benchmark sets generated from adenosine, camphor, cholesterol, limonene, and pyridine.

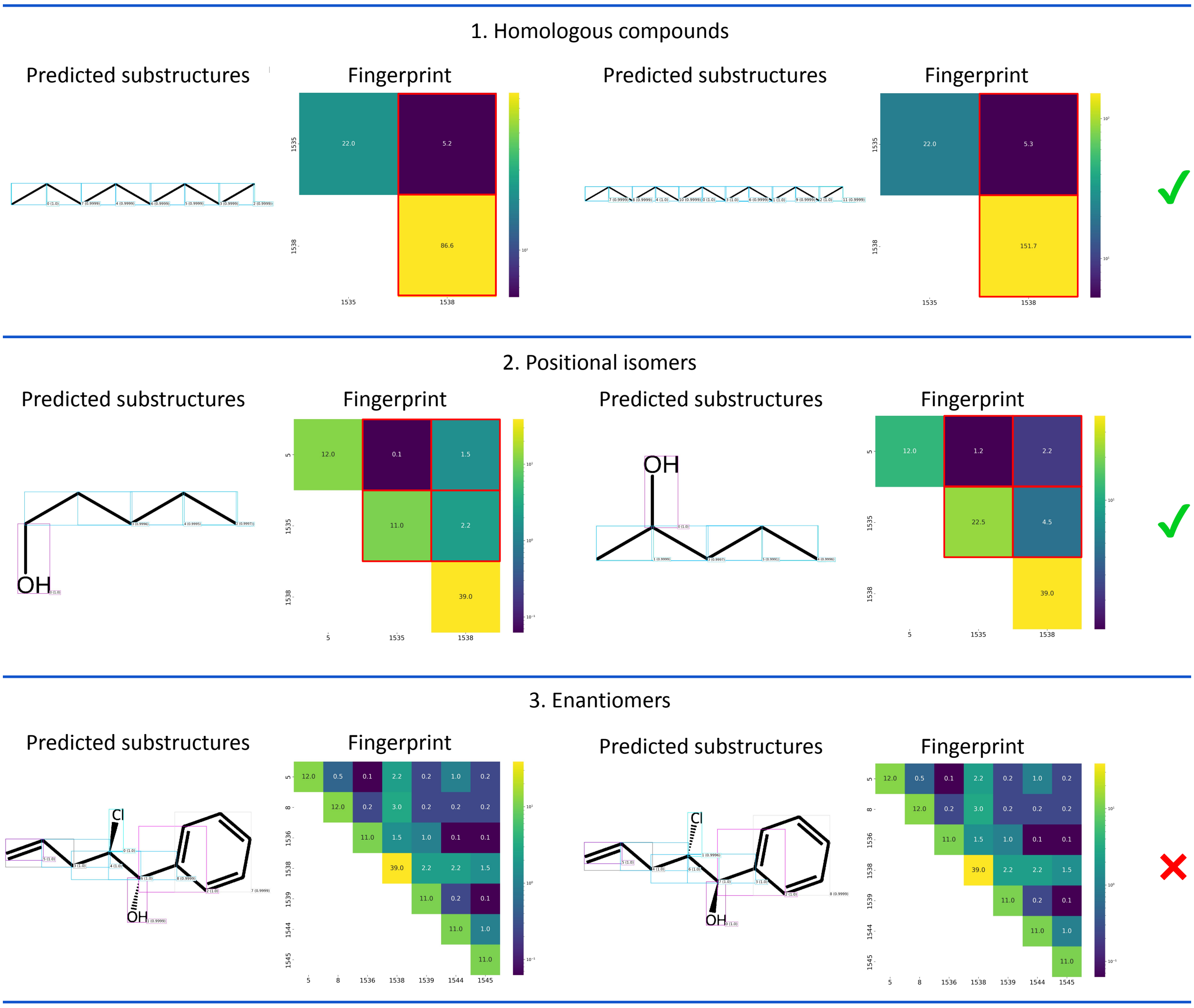


**Supplementary Figure 4**: **SVMF fingerprint discriminative capacity.** SubGrapher's predictions and SVMF fingerprints for example images representing (1) homologous compounds, (2) positional isomers, and (3) enantiomers. Cells in the SVMF fingerprints that differ between compounds are highlighted in red. SubGrapher distinguishes homologous compounds and positional isomers, but not enantiomers, since the substructures it detects lack stereochemistry information.

| Augmentation level | Adenosine | Camphor | Cholesterol | Limonene | Pyridine |
|---|---|---|---|---|---|
| Level 1 | 52 | 50 | 76 | 60 | 86 |
| Level 2 | 110 | 73 | 106 | 81 | 103 |
| Level 3 | 159 | 86 | 157 | 85 | 171 |

**Supplementary Table 3**: **Benchmarks augmentation analysis.** We compare the retrieval performance of SubGrapher on different variants of the image datasets generated from adenosine, camphor, cholesterol, limonene, and pyridine. Each variant uses different augmentations levels as described in the Supplementary Note 2. We report the average rank at which a query molecule is retrieved across 50 queries.

## Supplementary Note 2

To analyze the molecule retrieval results presented in the main manuscript, we perform evaluations on multiple augmented versions of the benchmarks. Supplementary Table 3 compares the retrieval performance of SubGrapher on different variants of the image datasets generated from adenosine, camphor, cholesterol, limonene, and pyridine. Each benchmark is augmented using different levels of augmentations. Level 1 corresponds to applying the augmentations:

- Rotation with factor drawn between -0.1 and 0.1 (applied with a probability of 90%),
- Scaling with factor drawn between -0.4 and -0.3 (90%),
- Downscaling with a factor drawn between 0.5 and 0.8 (70%),
- Grid distortion with factor drawn between -0.1 and 0.1 (50%).

Level 2 is used in the main manuscript and corresponds to applying the augmentations:

- Rotation with factor drawn between -0.1 and 0.1 (90%),
- Scaling with factor drawn between -0.7 and -0.5 (90%),
- Downscaling with a factor drawn between 0.7 and 0.99 (70%),
- Grid distortion with factor drawn between -0.15 and 0.15 (50%).

Level 3 corresponds to applying the augmentations:

- Rotation with factor drawn between -0.15 and 0.15 (90%),
- Scaling with factor drawn between -0.8 and -0.6 (90%),
- Downscaling with a factor drawn between 0.8 and 0.99 (70%),
- Grid distortion with factor drawn between -0.2 and 0.2 (50%).

We observe that augmentation, particularly strong downscaling and grid distortion, has a significant impact on performance. This effect is especially pronounced for the benchmarks derived from adenosine, cholesterol, and pyridine. An explanation is that these benchmarks contain more heteroatoms than the limonene and camphor benchmarks, making them more sensitive to the loss of detail caused by downscaling.

## Supplementary References


[1] Landrum, G. RDKit: Open-Source Cheminformatics Software. http://www.rdkit.org/. (Accessed: January 2025).

[2] Probst, D. & Reymond, J.-L. A probabilistic molecular fingerprint for big data settings. Journal of Cheminformatics **10**, 66 (2018).
